# Postbiotic Modulation of Retinoic Acid Imprinted Mucosal-like Dendritic Cells by Probiotic *Lactobacillus reuteri* 17938 *In Vitro*

**DOI:** 10.3389/fimmu.2016.00096

**Published:** 2016-03-17

**Authors:** Yeneneh Haileselassie, Marit Navis, Nam Vu, Khaleda Rahman Qazi, Bence Rethi, Eva Sverremark-Ekström

**Affiliations:** ^1^Department of Molecular Biosciences, The Wenner-Gren Institute, Stockholm University, Stockholm, Sweden; ^2^Department of Medicine, Karolinska University Hospital, Stockholm, Sweden

**Keywords:** retinoic acid, postbiotics, *Lactobacillus reuteri*, dendritic cells, probiotics

## Abstract

Lactobacilli are widely used as probiotics with beneficial effects on infection-associated diarrhea, but also used in clinical trials of e.g., necrotizing enterocolitis and inflammatory bowel diseases. The possibility of using probiotic metabolic products, so-called postbiotics, is desirable as it could prevent possible side effects of live bacteria in individuals with a disturbed gut epithelial barrier. Here, we studied how *Lactobacillus reuteri* DSM 17938 cell-free supernatant (*L. reuteri*-CFS) influenced retinoic acid (RA)-driven mucosal-like dendritic cells (DC) and their subsequent effect on T regulatory cells (Treg) *in vitro*. RA clearly imprinted a mucosal-like DC phenotype with higher IL10 production, increased CD103 and CD1d expression, and a downregulated mRNA expression of several inflammatory-associated genes (NFκB1, RELB, and TNF). Treatment with *L. reuteri*-CFS further influenced the tolerogenic phenotype of RA-DC by downregulating most genes involved in antigen uptake, antigen presentation, and signal transduction as well as several chemokine receptors, while upregulating IL10 production. *L. reuteri*-CFS also augmented CCR7 expression on RA-DC. In cocultures, RA-DC increased IL10 and FOXP3 expression in Treg, but pre-treatment with *L. reuteri*-CFS did not further influence the Treg phenotype. In conclusion, *L. reuteri*-CFS modulates the phenotype and function of mucosal-like DC, implicating its potential application as postbiotic.

## Introduction

The mucosal immune system in the gut is continuously exposed to innocuous dietary antigens and commensal microbes. Apart from its well-known effects on gut homeostasis and metabolic functions, this local microenvironment plays a major role in shaping the response of immune cells in their specialized tissue-associated functions; for instance intestinal epithelial cells influence the gut dendritic cells (DC) response, which subsequently influence T cell responses. Factors such as thymic stromal lymphopoietin and indoleamine 2,3-dioxygenase produced by epithelial cells confer a tolerogenic phenotype on DC (1). Epithelial cells have also been shown to metabolize vitamin A into retinoic acid (RA) (2). Among its pleiotropic effects, RA has been shown to promote the migration of pre-DC, the precursor of CD103^+^ DC, to the small intestine (3) and to induce CD103^+^ expression in monocyte derived (Mo-) DC *in vitro* (4). Coculturing RA-DC with T cells induces upregulation of the gut homing markers CCR9 and α4β7, and constitutive production of IL10 in T cells (5, 6). But the response of RA-DC to gut microbes has not yet been evaluated *in vitro*.

Although usually present in low numbers, lactobacilli are common inhabitants of the human gut, in particular in early life (7). Lactobacilli seem capable of modulating both mucosal and systemic immune responses, but the effects on the immune system vary between strains (8), and some lactobacilli are able to induce the production of both pro-inflammatory and regulatory cytokines, such as IL6, TNF-α, and IL10 (9, 10). Due to their generally accepted health benefits, lactobacilli are widely consumed as probiotics and their therapeutic potential has been considered for future treatment of several disorders (11–15).

One of the most commercially and clinically recognized *Lactobacillus* strain is *Lactobacillus reuteri* DSM 17938 (*L. reuteri* 17938) (16). *L. reuteri* DSM 17938 is derived from *L. reuteri* ATCC 55730, isolated from a Peruvian mother’s breast milk. Two plasmids harboring antibiotic resistance genes were removed from strain 55730 to obtain strain 17938, which inhibits pathogen growth and modulates the immune system (17). It has been shown that feeding *L. reuteri* DSM 17938 to newborn rats in an animal model of necrotizing enterocolitis (NEC) induced a strong anti-inflammatory effect through inhibiting TLR4 signaling pathway in the intestine (18). This resulted in a reduction of the severity of the disease by modulation of the balance between T effector/memory (Tem) cells and regulatory T (Treg) cells (19).

Although most of its effects require direct bacteria–host cell contact, some lactobacilli-produced soluble factors have also been shown to modulate the immune response. Understanding the mechanisms by which soluble factors produced by probiotics, recently termed postbiotics, modulate immune function may be vital to develop alternative therapeutic techniques (20). The aim of this study was to elucidate the influence of *L. reuteri* 17938 cell-free supernatant (CFS) on the response of RA-DC. We further investigated how RA-treatment of DC as well as *L. reuteri*-exposure influenced subsequent T cell responses.

## Materials and Methods

### *Lactobacillus reuteri* DSM 17938

A CFS from *Lactobacillus* (*L*.) *reuteri* DSM 17938 (Biogaia AB, Stockholm, Sweden) was kindly provided by Stefan Roos, the Swedish University of Agricultural Sciences. *L. reuteri* DSM 17938 was cultured in MRS broth (Oxoid, Hampshire, UK) at 37°C for 20 h (still culture). The CFS was removed from the bacterial pellet by centrifugation at 14,000 *g*. The supernatant was sterile filtered (0.2 μm) and stored at −20°C until used. Before stimulation of DC, the *L. reuteri*-CFS was diluted 1:1 with HEPES to keep the pH at 7.2–7.5 (21).

### Generation and Activation of Immature RA-DC and Mo-DC from CD14^+^ Monocytes

Peripheral blood mononuclear cells (PBMC) were isolated from buffy coats from healthy blood donors using Ficoll (GE Healthcare, Logan, UT, USA) gradient centrifugation. CD14^+^ monocytes were enriched by negative selection using EasySep™ human monocyte enrichment kit (STEMCELL™ Technologies, Grenoble, France) according to the manufacturer’s protocol. The 4 × 10^5^ cells/ml of the enriched monocytes were seeded in 6-well plates in complete culture medium (RPMI-1640, GE Healthcare), 10% heat-inactivated fetal bovine serum (Gibco-Invitrogen, Waltham, MA, USA), 1% l-glutamine (GE Healthcare), 1% penicillin–streptomycin (Thermo Scientific, Waltham, MA, USA), 2% sodium pyruvate (Gibco-Invitrogen), 2% HEPES buffer (GE Healthcare), and 50 μM β-mercaptoethanol (Sigma-Aldrich, St Louis, MO, USA), supplemented by 25 ng/ml rHuGM-CSF and 20 ng/ml rHuIL4 (both from Peprotech Inc., Rocky Hill, NJ, USA). To generate RA-DC, 1.15 μg/ml RA (Sigma-Aldrich) was also added once to the monocyte cultures. On day 3, the cultures were refreshed with new media, supplemented with IL4, GM-CSF, and RA.

On day 6, RA-DC and Mo-DC were further stimulated with 5% *L. reuteri*-CFS for 24 h. As controls, cells were stimulated with ultrapure LPS-0111:B4 (500 ng/ml) (InvivoGen, San Diego, CA, USA) or were kept in culture medium. Culture supernatants from the 24 h-stimulations were collected and stored at −20°C until further analysis. The cells were used for surface staining.

All experiments were approved by the Regional Ethics Committee in Stockholm (Dnr 2014/2052-32). All study subjects gave their informed written consent, and all samples were coded and stored and used as stated in the approved ethical application. It is not possible to connect published data to any individual.

### Gene Expression Profiling of RA-DC and Mo-DC Using RT^2^ Profiler™ PCR Array

Gene expression profile in Mo-DC and RA-DC was investigated by using the RT^2^ Profiler™ PCR Array Human Dendritic and Antigen Presenting Cell (PAHS-406ZF) (Qiagen, Germantown, MD, USA) following stimulation with *L. reuteri*-CFS or LPS for 24 h. The 1.50 × 10^6^ Stimulated DC cells were homogenized with QIAshredder^®^ (Qiagen) and total RNA was isolated with the RNeasy^®^ Mini Kit (Qiagen). The amount, purity, and quality of the generated RNA were evaluated using a NanoDrop™ 8-sample spectrophotometer (Thermo Scientific) and an Agilent 2100 Bioanalyzer (Agilent Technologies, Santa Clara, CA, USA). Only RNA samples with a RNA integrity number (RIN) > 8.5 were used for gene expression analysis. The RT^2^ First Strand Kit (Qiagen) was used to generate cDNA, starting with 0.5 μg of total RNA for each sample. Quantitative polymerase chain reactions were performed using the RT^2^ SYBR Green ROX qPCR Mastermix as recommended by the manufacturer (Qiagen), and the array was run with the LightCycler^®^ 480 real-time PCR (Roche Applied Science, Penzberg, Germany). Results were analyzed in the web-based GeneGlobe Data Analysis Center (Qiagen). Gene expression levels were normalized to the reference genes: ribosomal protein, large, P0 (RPLPO), and glyceraldehydes-3-phosphate dehydrogenase (GAPDH). The reference genes were selected based on their stability in expression among samples (*C*_T_ difference <1).

### Staining and Flow Cytometry Analysis of RA-DC and Mo-DC

RA-DC and Mo-DC were stained with the following monoclonal antibodies: CD14 FITC-A (clone M5E2), CD1d PE-A (clone CD1d42), CD86 PerCP-A (clone IT2.2), CD83 PE-Cy7-A (clone HBI5e), CD11c APC-A (clone B-ly6), CD103 PE-A (clone Ber-ACT8), HLA-DR PerCP-A (clone G46-6), DC-SIGN BV421-A (clone DCN46) (all from BD Biosciences, San Jose, CA, USA), and CCR7 BV421-A (clone G043H7) (BioLegend, San Diego, CA, USA). The FACSVerse instrument and the FACS Suite software (BD Biosciences) were used to acquire data. Corresponding isotype-matched antibodies were used as negative controls. Detailed gating strategies for the DC can be found in Figure S1A in Supplementary Material. The results show either the percentage of positive cells within a given population or the mean surface expression of receptors per cell defined as geometrical mean fluorescence intensity (MFI). Analysis was done with FlowJo Software (TreeStar, Ashland, OR, USA) and Cytobank (Cytobank, Inc., Mountain View, CA, USA).

### Cytokine Analysis (ELISA)

Levels of IL6, IL10, IL12, IL23, TGFβ1, and TNFα in the DC culture medium were measured with enzyme-linked immunosorbent assays (ELISA) kits (MabTech, Nacka, Sweden). In short, plates were coated for 24 h, washed, and blocked with BSA 0.1% v/v in PBS with 0.05% Tween. The DC supernatant was added and incubated overnight. Biotin-labeled secondary antibodies and streptavidin-ALP were then added prior to the phosphate substrate (Sigma-Aldrich). The optical density was determined using a micro-plate reader (Molecular Devices Corp., Sunnyvale, CA, USA) set at 405 nm. Results were analyzed using SoftMax Pro 5.2 rev C (Molecular Devices Corp.).

### Chemokine Array

Human chemokine array kit (Abcam, Cambridge, UK) was used to detect 38 chemokines simultaneously in supernatants from Mo-DC and RA-DC after stimulation. The antibody-coated nitrocellulose membranes were incubated with blocking buffer for 30 min. DC-supernatants from each sample were diluted 1:1 with blocking buffer, added to the membranes and incubated on a rocking platform at 2–8°C overnight. After washing the membranes, a cocktail of biotin-conjugated anti-chemokine antibodies was added in to each membrane and incubated for 2 h, followed by repetitive washing. After 2 h of incubation with streptavidin-HRP, each nitrocellulose membrane was rinsed and exposed to detection buffer for 1 min to develop chemiluminescent spots. Images were captured using a luminescent image analyzer (Fujifilm, LAS-100 plus, Tokyo, Japan). In order to quickly identify the positive signals on developed image, a transparent overlay template was placed on the array image film and aligned with the pairs of reference spots in each array. The spot intensity was qualitatively compared among the groups.

### T Cells Coculture with RA-DC and Mo-DC Pre-Conditioned with *L. reuteri*-CFS

RA-DC and Mo-DC were stimulated for 5 h with 5% *L. reuteri*-CFS. Prior to coculture with T cells, the stimulated DC were washed three times with complete culture medium to prevent carry-over of *L. reuteri*-CFS to the coculture. Autologous T cells were negatively selected from PBMC with the EasySep™ Human T Cell Enrichment Kit (STEMCELL™ Technologies) according to the manufacturer’s instructions. T cell purity was 95.9% as determined by flow cytometry. DC and T cells were cocultured in a 1:4 ratio (DC:T cell) for 24 h without additional stimuli. The cells were used for flow cytometry analysis.

### Staining and Flow Cytometry Analysis of T Cells

Following coculture with RA-DC and Mo-DC, T cells were stained for surface and intracellular markers using CD3 Pecy7-A (clone SK7), CD25 BV421-A (clone BC96) and IL10 APC-A (clone JES3-pD7) (all from BioLegend), CD4 FITC-A (clone RPA-T4), CD8 APC-H7-A (clone SK1), and FoxP3 PE-A (clone 259D/C7) (all from BD Biosciences). Live/dead fixable dead cell stain kit Aqua-A (Life Technologies, Eugene, OR, USA) was included in all panels. For the intracellular staining of FoxP3 and IL10, monensin (BD Biosciences) was added to the coculture 5 h before analysis and then cells were fixed and permeabilized prior to intracellular staining. Corresponding isotype-matched antibodies were used as negative controls. Detailed gating strategies can be found in Figure S1B in Supplementary Material. The FACS analysis was performed as mentioned above.

### Statistics

To assess differences between immature Mo-DC and immature RA-DC or between stimulated and the unstimulated DC, the obtained raw data was log-transformed. The normal distribution of the log transformed was tested using D’Agostino–Pearson Omnibus or Shapiro–Wilk normality test. If the data pass the normality test, the log-transformed data were analyzed with one-way ANOVA. If differences among groups were significant, Bonferroni multiple comparisons-test was performed to control for multiple comparisons and confirm the *p*-values between paired groups. If the data were not normally distributed, Kruskal–Wallis ANOVA was used to investigate differences among the groups. When significant, the Mann–Whitney *U*-test was further used to investigate differences between groups. All statistical computations were performed using GraphPad Prism software V6 (La Jolla, CA, USA).

## Results

### Enhanced Expression of Gut Homing-Associated Genes and Reduced Expression of Genes Associated with Inflammation in RA-DC

**Figure 1:**
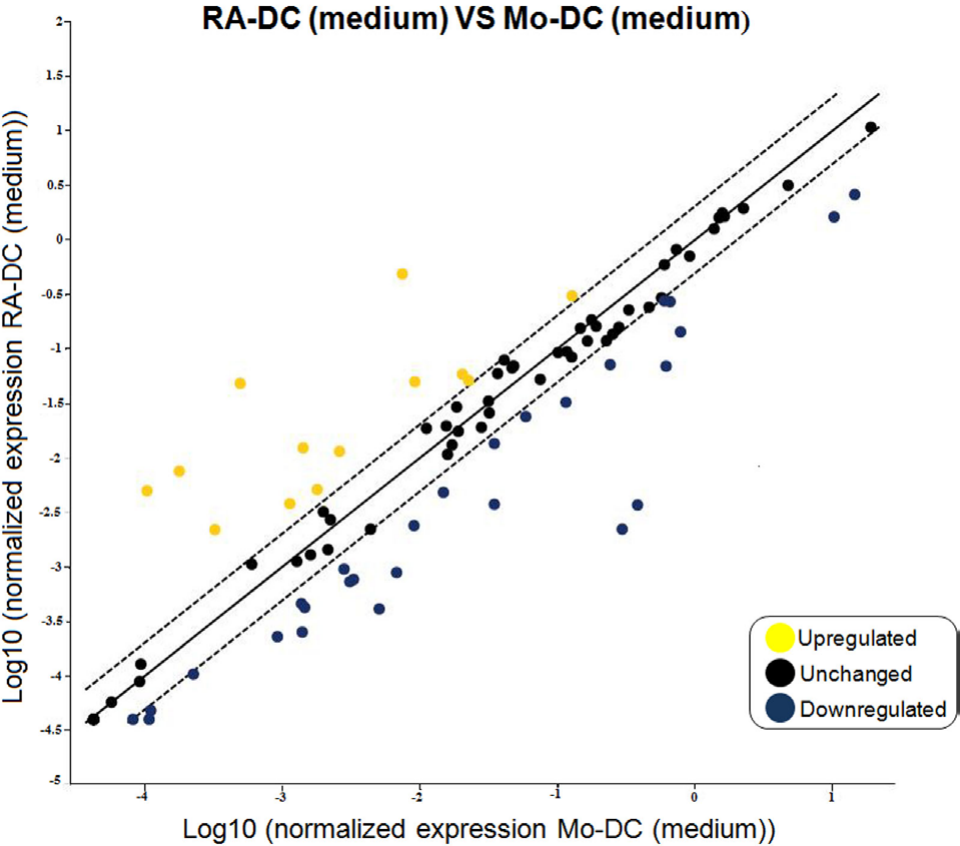
**RA treatment imprints down-regulation in gene expression of dendritic cells**. RT^2^ PCR arrays were performed on the RNA extracted from RA-DC and Mo-DC at day 6 of culture. Scatter plots shows the fold change of the gene expression of the RA-DC in culture medium in comparison to that of the Mo-DC in culture medium. Upregulated genes are shown in yellow, downregulated genes are shown in blue, and unchanged (less than twofold change) genes in black.

To unravel the broad impacts of RA on the DC phenotype, we measured the expression of an array of DC-associated genes in RA-DC and Mo-DC. Genes for IL6, IL10, and CD1d were upregulated in the RA-DC population. Apart from this, most other genes that differed in expression between RA-DC and Mo-DC genes were less expressed in RA-DC (Figure 1, Table S1 in Supplementary Material). There was a downregulation of mRNA expression for several factors in RA-DC with key functions in an inflammatory setting, like IL12b, IFNG, TNF, RELB, and NFκB1. Further, several genes associated with antigen presentation and T cell interactions were downregulated, like CD74, CD80, HLA-DMA, HLA-DPA, CD1A, CD1B, and CD1C. Gene expression of molecules associated with transmigration and gut homing like ICAM1 and ICAM2 were upregulated in RA-DC together with some chemokines such as CCL2, CCL5, and CCL7. Still, the expression of other chemokines, including CCL19, CXCL1, CXCL10, and CXCL2 was downregulated. In addition, RA-DC had a lower expression of the chemokine receptors CXCR1, CCR2, and CXCR4.

### RA Treatment Increases the Percentage of CD14 Expressing CD11c^+^ DC

**Figure 2:**
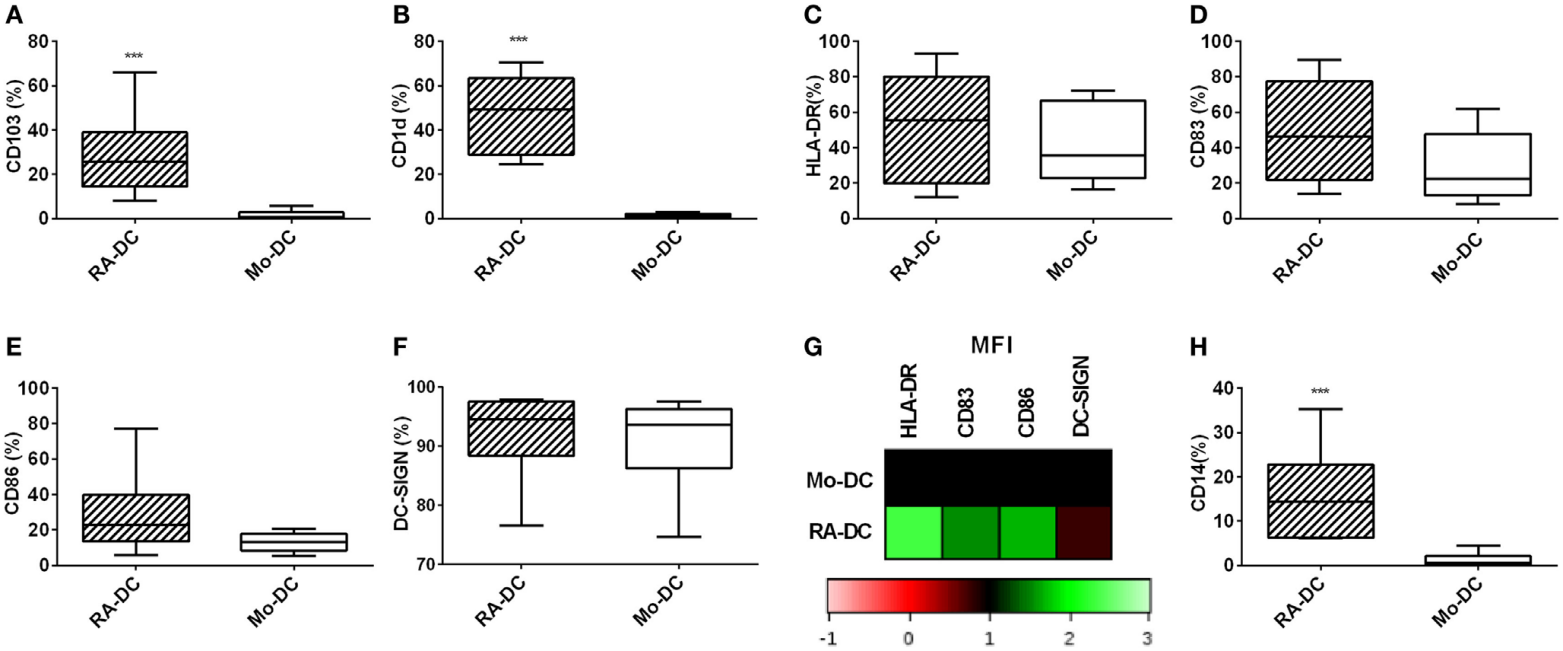
**RA treatment increases the percentage of CD14 expressing CD11c^+^ DC**. Box plot graphs **(A–F)** show the percentage of CD11c^+^ cells expressing CD103 **(A)** CD1d **(B)**, HLA-DR **(C)**, CD83 **(D)**, CD86 **(E)**, and DC-SIGN **(F)** within the RA-DC and Mo-DC populations at day 6. Representative heat map showing MFI fold changes of the surface markers HLA-DR, CD83, CD86, and DC-SIGN of CD11c^+^ RA-DC in relation the MFI of the Mo-DC is shown in **(G)**. **(H)** shows a box plot graph of percentage of CD14-expressing cells in CD11c^+^ RA-DCs and Mo-DC. *N* ≥ 6, ***P < 0.001, **P < 0.01, and *P < 0.05.

To further evaluate the phenotype of these DC at a protein level, we investigated the RA-DC and Mo-DC regarding expression of surface proteins associated with DC differentiation, functional maturation, and a mucosal DC phenotype. Within the CD11c^+^ DC population, there were marked differences in CD103 and CD1d expression between RA-DC and Mo-DC, with significantly higher percentages of positive cells among the RA-DC population, indicative of a mucosal-DC like phenotype of RA-DC (Figures 2A,B). While both types of DC expressed similar percentages of HLA-DR, CD83, CD86, and DC-SIGN positive cells (Figures 2C–F), there was a markedly higher expression (MFI) of these markers in the RA-DC, except for DC-SIGN (Figure 2G and Table S2 in Supplementary Material). Notably, the percentage of CD14 positive cells in the CD11c^+^ population was significantly higher in the RA-DC (Figure 2H).

### RA Treatment Induces IL10 Production and Increases Secretion of CCR2 Ligands, but Decreases GRO and CCL23

**Figure 3:**
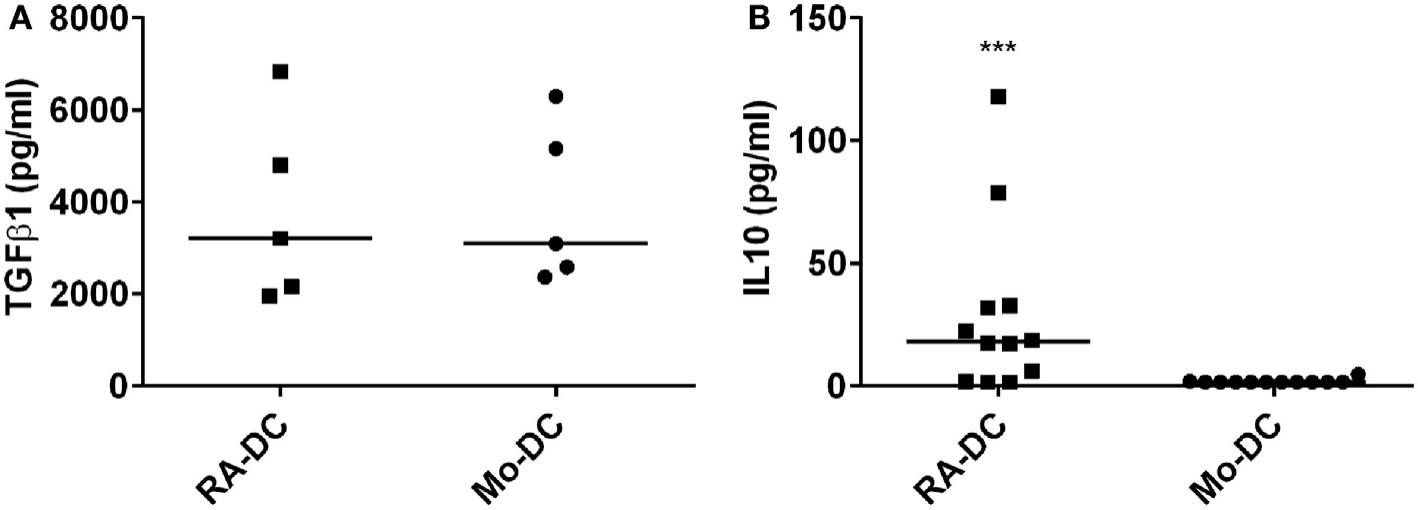
**RA treatment induces IL10 production**. Spontaneous secretion of TGFβ1 **(A)** and IL10 **(B)** in the DC culture supernatants at day 6, as measured by ELISA. *N* = 4–12, ***P < 0.001, **P < 0.01, and *P < 0.05.

To investigate the influence of RA on the secretory profile of these DC, we measured spontaneously secreted cytokine and chemokines levels in the RA-DC and Mo-DC culture supernatants, with ELISA and chemokine arrays, respectively. TGFβ1 was highly detected in both types of DC (Figure 3A), while the level of IL10 in the RA-DC culture supernatant was significantly higher than that of the Mo-DC (Figure 3B). The levels of IL6, IL12, IL23, and TNFα were below the detection limit for both types of DC (data not shown).

Among 38 chemokines investigated, chemokines such as CXCL8, CXCL10, CCL13, CCL3, and CCL5 were constitutively detected in both RA-DC and Mo-DC supernatants (Table 1). In RA-DC supernatants, CCL2, CCL8, and CCL7 were detected in higher intensity, while GRO and two isoform of CCL23 (Ckβ8-1 and MPIF-1) were detected in lower intensity in comparison to Mo-DC.

**Table 1:** **Chemokine (protein) profiling of supernatants from RA-DC and Mo-DC stimulated with *L. reuteri* 17938-CFS**.

	RA-DC medium	RA-DC *L. reuteri*	Mo-DC medium	Mo-DC *L. reuteri*
CCL23	+	+	++	++
CCL27	+	+	+	+
CXCL16	+	+	+	+
CXCL5		++		++
CCL24	+	+	+	+
CCL26	+			
CXCL6		+		
GRO	+	++	++	++
CXCL1		++		++
CCL1				++
CXCL8	++	++	++	++
CXCL10	+	+	+	+
CCL2	++	++	+	++
CCL8	++	++	+	+
CCL7	++	++		
CCL13	+	+	+	+
CCL22	+	+	+	+
CCL3	+	++	+	++
CCL4	+	+	+	+
CCL15		+		+
CCL20		++		+
CCL23			++	+
CXCL7	+	+	+	+
CCL5	++	++	++	+
CCL17	+	+	+	+

*^+^a spot with lower density than reference spots*.

*^++^a spot with similar or greater density than reference spots*.

*Blank space means undetected*.

### *L. reuteri*-CFS Has a Strong Influence on Gene Expression in RA-DC and Mo-DC

**Figure 4:**
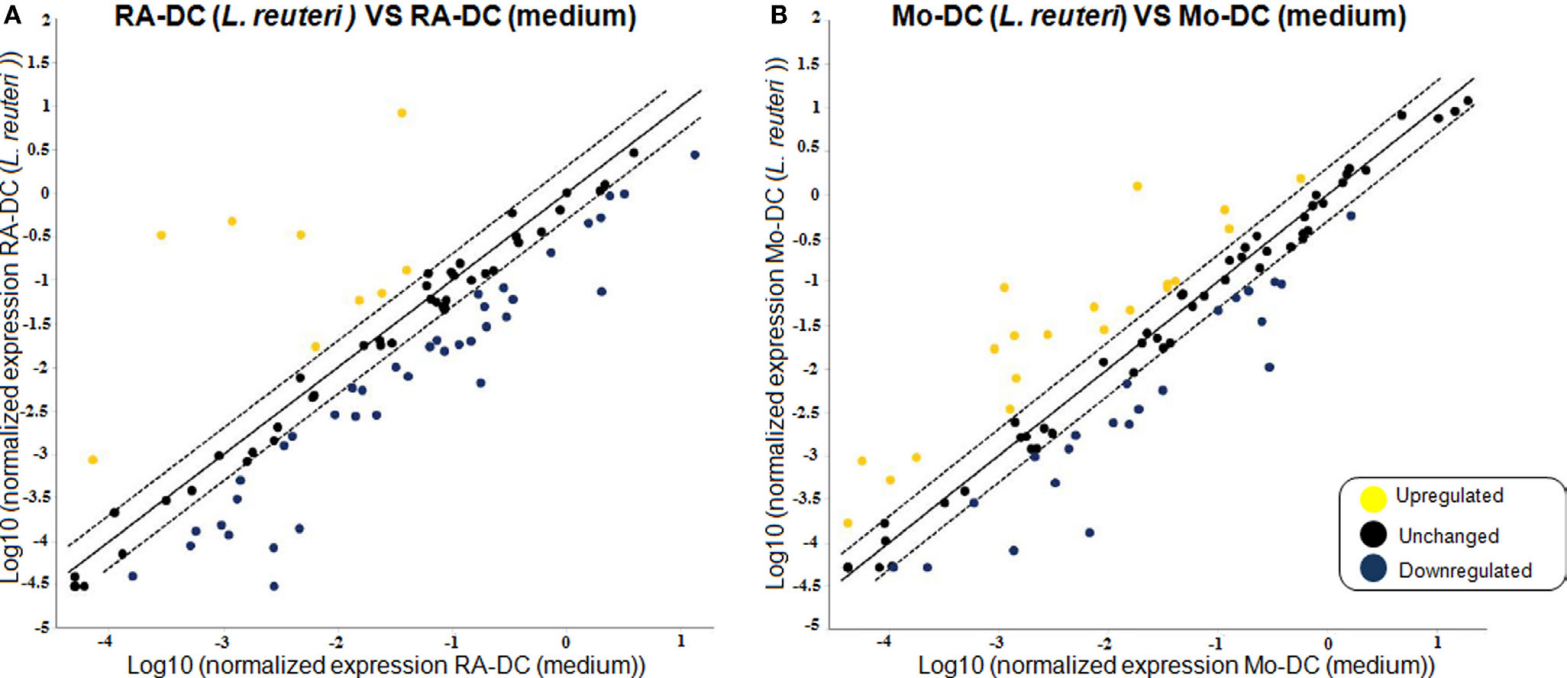
***L. reuteri*-CFS differentially influences the expression of genes in RA-DC and Mo-DC**. RT^2^ PCR arrays were performed on the RNA extracted from the RA-DC and Mo-DC treated with *L. reuteri*-CFS for 24 h. Scatter plots show the fold change of the gene expression of the *L. reuteri 17938* treated RA-DC **(A)** and Mo-DC **(B)** in comparison to culture medium. Upregulated genes are shown in yellow, downregulated genes are shown in blue, and unchanged (less than twofold change) genes in black.

To elucidate whether postbiotics derived from *L. reuteri* DSM 17938 could influence the functional phenotype and secretory profile of RA-DC and Mo-DC, we investigated the expression of DC-associated genes post-stimulation with *L. reuteri*-CFS. In RA-DC, *L. reuteri-*CFS induced a strong upregulation of a few genes, while most of the investigated genes were downregulated in comparison to unstimulated RA-DC (Figure 4A). In contrast, the *L. reuteri*-CFS stimulated Mo-DC showed evenly distributed upregulated and downregulated genes in comparison to unstimulated Mo-DC (Figure 4B). Few upregulated genes in *L. reuteri*-treated RA-DC were chemokines (CCL5, CXCL1, CXCL2, and CXCL8) and cytokines (IL6 and IL10), together with the genes for thrombospondin 1 (THBS1) and colony stimulating factor 2 (CSF2) (Table S3 in Supplementary Material). Gene expression for several chemokines (CCL13, CCL19, CXCL10, and IL16), chemokine receptors (CCR1, CCR2, CXCR1, and CCR5), and molecules involved in antigen presentation and DC-T cell cross talk (CD1A, CD1B, CD1C, and CD1D, HLA-DMA, HLA-DPA1, CD74, CD40) were downregulated. Lastly, RA-DC stimulated with *L. reuteri*-CFS also downregulated cell adhesion molecules (VCAM1, ICAM2, ITGAM, and ITGB2) and transcription factors (IRF8 and CEBPA).

Mo-DC responded in a partly different way to *L. reuteri*-CFS with an increase in the mRNA expression of molecules associated with T cell contact like CD1D, CD80, CD86, and IL12B (encoding for the p40 subunit of IL12 and IL23) (Table S3 in Supplementary Material). Notably also the expression of FCGR1A (CD64) and FCER1A (FcεRI) differed from that of RA-DCs, with a remarkable up- and down-regulation, respectively.

For comparison, we also evaluated how *L. reuteri*-CFS influenced the gene expression of these two DC populations in comparison with LPS, which is frequently used to mature DC.

As depicted in the clustogram, the gene expression profile induced by *L. reuteri*-CFS is distinctly different from that following LPS exposure, and this was most evident for RA-DC (Figure S2 in Supplementary Material). Further, in their gene expression profile, the RA-DC show a more dampened response to LPS compared to that of the Mo-DC (Figure S3 in Supplementary Material).

### *L. reuteri*-CFS Upregulates CD14 and Downregulates DC-SIGN Expression on RA-DC, Without Affecting Activation Markers

**Figure 5:**
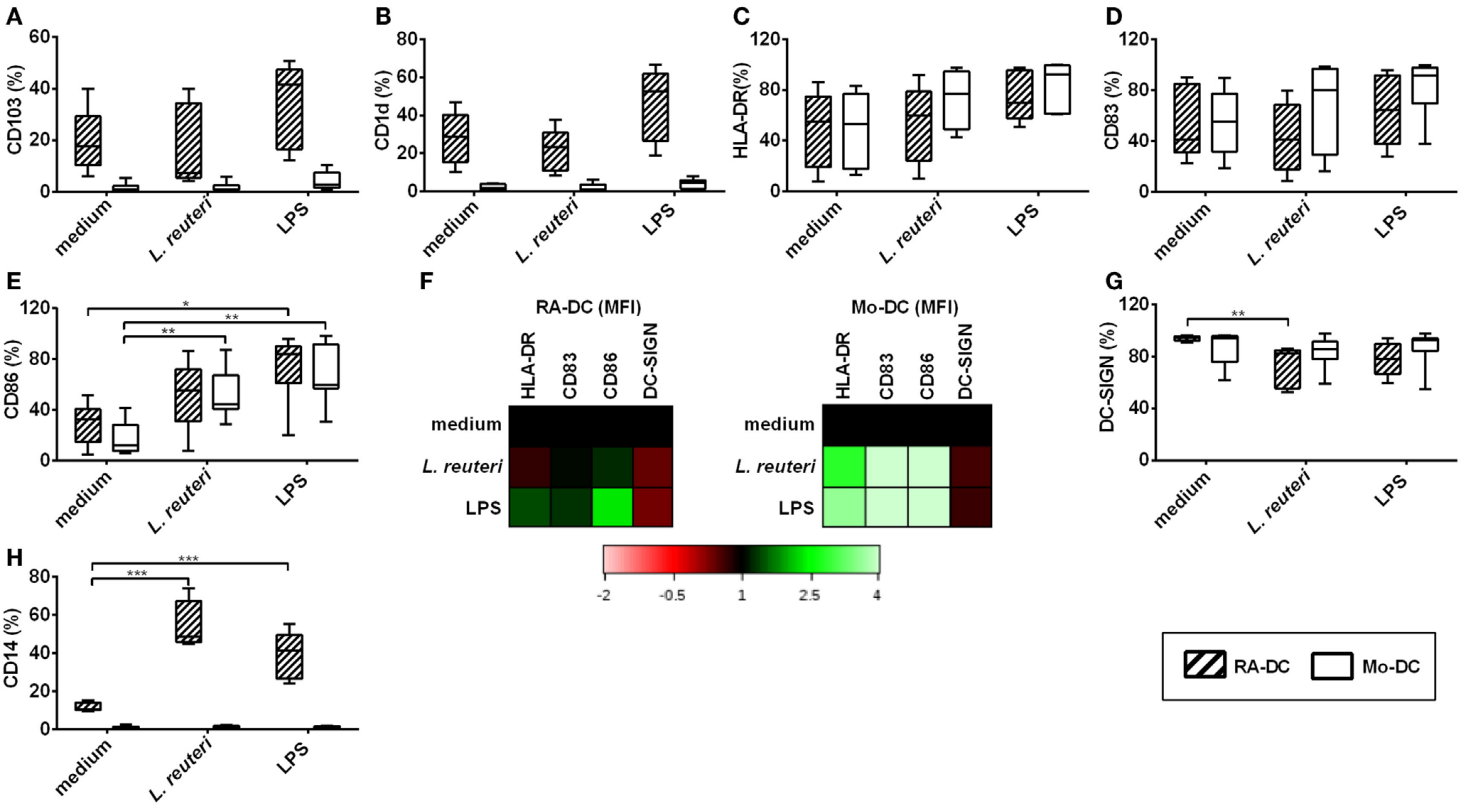
***L. reuteri*-CFS upregulates CD14 and downregulates DC-SIGN expression on RA-DC**. RA-DC and Mo-DC were treated with *L. reuteri*-CFS or LPS or for 24 h. Box plot graphs **(A–E)** show the percentage of CD11c^+^ cells expressing CD103 **(A)**, CD1d **(B)**, HLA-DR **(C)**, CD83 **(D)** and CD86 **(E)** in RA-DCs and Mo-DC post-treatment. Representative heat map showing MFI fold changes of the surface markers HLA-DR, CD83, CD86, and DC-SIGN of CD11c^+^ RA-DC and Mo-DC post-treatment in comparison to culture medium is shown in **(F)**. **(G,H)** shows the percentages of DC-SIGN^+^ **(G)** and CD14^+^ **(H)** CD11c^+^ cells in RA-DC and Mo-DC populations post-treatment. *N* ≥ 5, ***P < 0.001, **P < 0.01, and *P < 0.05.

We further investigated the effect of *L. reuteri*-CFS on the expression of surface proteins on both RA-DC and Mo-DC. *L. reuteri*-CFS did not influence the expression of CD103, CD1d, HLA-DR, or CD83 (Figures 5A–D), while it increased the percentage of CD86 positive cells in Mo-DC (Figure 5E). The MFI of markers associated with DC activation (HLA-DR, CD83, and CD86) increased for Mo-DC treated with *L. reuteri*-CFS, but not for *L. reuteri*-CFS-treated RA-DC (Figure 5F, Table S4 in Supplementary Material). On the other hand, the percentage of DC-SIGN significantly decreased in the RA-DC upon stimulation (Figure 5G). Remarkably, *L. reuteri*-CFS further increased the percentage of CD14^+^ cells in the RA-DC, without influencing that of the Mo-DC (Figure 5H).

### *L. reuteri*-CFS Significantly Impacts the Secretory Profile of RA-DC and Mo-DC

**Figure 6:**
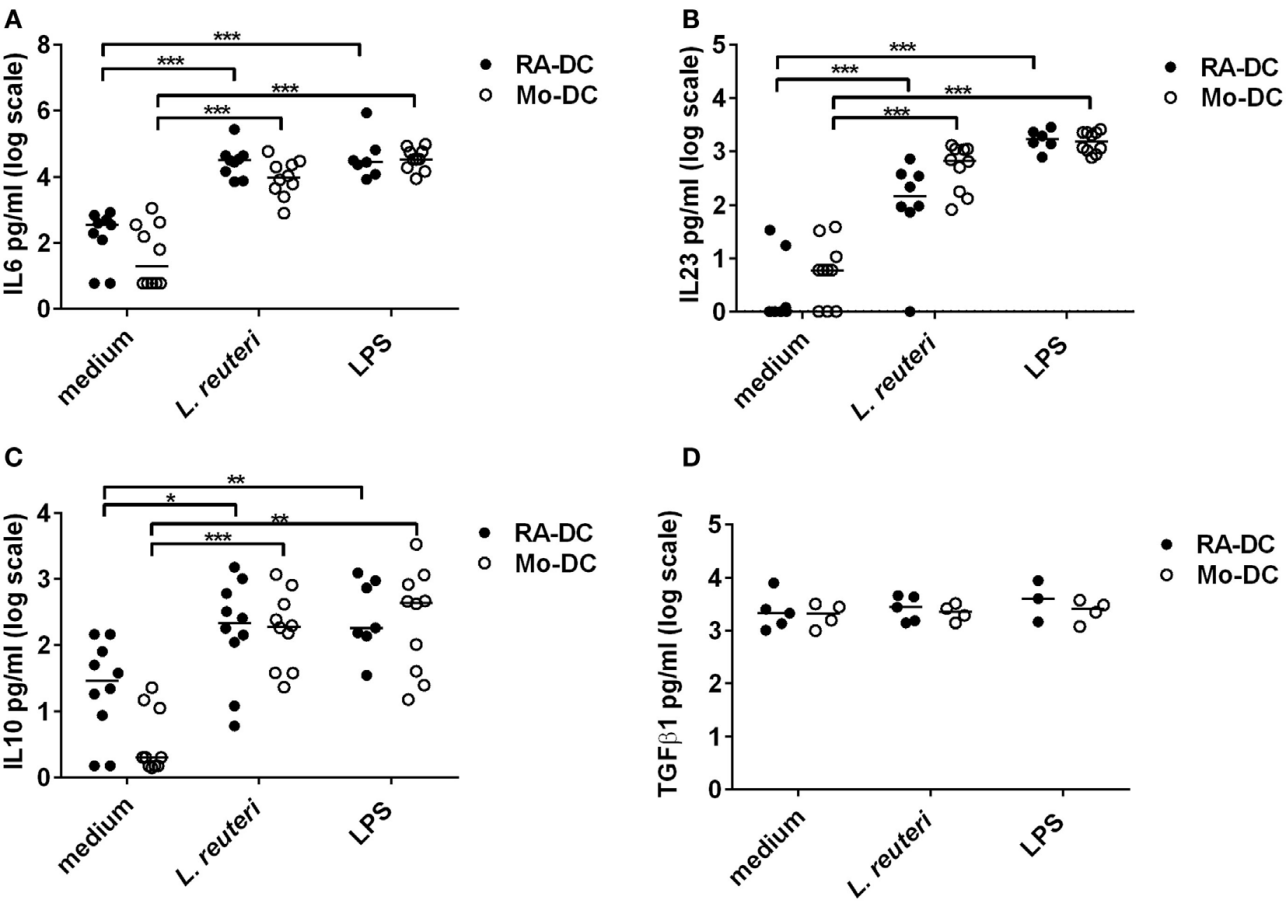
***L. reuteri*-CFS upregulates cytokine production in both RA-DC and Mo-DC**. RA-DC and Mo-DC were treated with *L. reuteri*-CFS or LPS for 24hrs. The production of IL6 **(A)**, IL23 **(B)**, IL10 **(C)**, and TGFb1 **(D)** from RA-DC and Mo-DC post-treatment was measured by ELISA. *N* = 4–7, ***P < 0.001, **P < 0.01, and *P < 0.05.

*Lactobacillus reuteri*-CFS was potent in inducing IL6, IL23, and IL10 by both RA-DC and Mo-DC (Figures 6A–C), while TGFβ1 levels did not change with *L. reuteri*-CFS stimulation (Figure 6D).

*L. reuteri*-CFS induced CXCL1, CXCL5, CCL3, CCL15, and CCL20 production in both RA-DC and Mo-DC (Table 1). Secreted CCL1 was only detected in Mo-DC after stimulation, while CCL7 was only produced in RA-DC but regardless of *L. reuteri* stimulation.

### *L. reuteri*-CFS Upregulates CCR7 Expression on RA-DC

**Figure 7:**
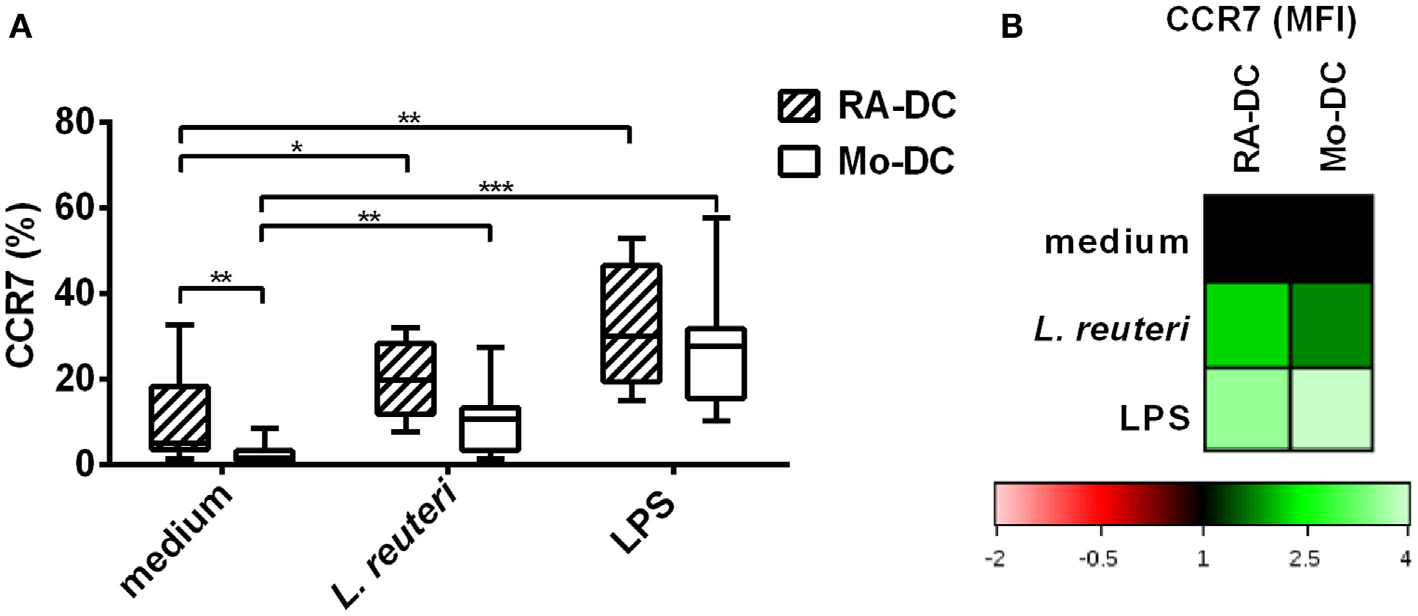
***L. reuteri*-CFS upregulates CCR7 expression on RA-DC**. RA-DC and Mo-DC were treated with *L. reuteri*-CFS or LPS for 24 h. The box plot graph shows the percentage of CCR7 expressing cells in CD11c^+^ RA-DCs and Mo-DC, post-treatment **(A)**. **(B)** Representative heat map showing MFI changes of CCR7 expression in CD11c^+^ RA-DC and Mo-DC post-treatment, compared to their respective DC in culture medium.

A hallmark of mucosal DC is the ability of these cells to migrate to the mesenteric lymph node (MLN) by upregulating the expression of CCR7. We investigated CCR7 expression on RA-DC and Mo-DC. Although treatment with RA by itself causes increased expression of CCR7 (Figure 7A, medium), stimulation with *L. reuteri*-CFS further upregulated the expression of CCR7 on the RA-DC both in terms of the percentage of positive cells (Figure 7A) and level of expression (MFI) [average 1.78 (1.36–2.26)] (Figure 7B).

### RA-DC-Induced FOXP3 and IL10 Expression in Treg Cells (CD25^+^ FOXP3^+^ CD4^+^ Cells)

**Figure 8:**
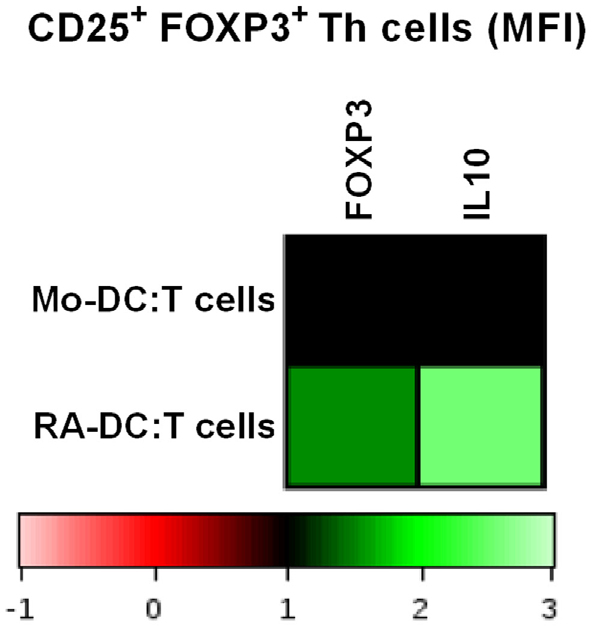
**RA-DC enhanced FOXP3 expression and IL10 production in Treg cells (CD25^+^ FOXP3^+^ Th cells)**. T cells were cocultured with RA-DC and Mo-DC for 24 h. Representative heat map showing the MFI of FOXP3 and IL10 in Treg cells (CD25^+^ FOXP3^+^ Th cells) cocultured with RA-DC and Mo-DC, respectively. *N* ≥ 3

Once mucosal DC migrate to the MLN, they interact with T cells. To investigate the influence of the generated DC in terms of their ability to promote a Treg response, we cocultured the RA-DC and Mo-DC with autologous T cells. For both RA-DC:T cells and Mo-DC:T cell cultures, the percentage of Treg cells (CD3^+^CD4^+^CD25^+^FOXP3^+^) and/or IL10^+^ Treg cells were similar (data not shown). However, upon coculture with RA-DC, Treg cells had an increased expression of FOXP3 and IL10 compared to when cocultured with Mo-DC (Figure 8) [FOXP3 MFI average increase 1.24 (1.08–1.54) and IL10 MFI average increase 1.74 (1.21–2.57) for RA-DC:T cell cultures]. Preconditioning of the RA-DC with *L. reuteri*-CFS did not further enhance the Treg response (data not shown).

## Discussion

In the human gut, the micro-milieu regulates DC to display a tolerogenic phenotype at steady state to prevent an inflammatory condition that could disturb the homeostasis. RA is believed to be of vital importance in this process, through the induction of CD103^+^ CX3CR1^−^ DC and eventually also Treg cells. Here, we generated CD103-expressing mucosal-like DC from human peripheral CD14^+^ monocytes, and thoroughly investigated their gene profile, functional- and secretory profile, and interaction with T cells. We report significant differences between RA-DC and Mo-DC, both on gene- and protein levels, with the RA-DC clearly having a more regulatory and tolerogenic profile. Further, we describe how *L. reuteri* DSM 17938-CFS, a commonly used probiotic strain, impacts the RA-DC and Mo-DC in different ways, adding to the tolerogenic profile of the RA-DC, but with little effect on Treg characteristics.

In general only few genes were upregulated in RA-DC compared with Mo-DC, while the majority of genes were downregulated. Among those genes that were significantly more expressed in the RA-DC were IL6 and IL10, confirmed also at protein level for IL10. To our knowledge, we are the first to show that RA-DC have a constitutive baseline production of IL10 when kept in culture medium without continuous RA-supplementation during the entire culture period. The expression of a majority of the investigated chemokine genes was downregulated in RA-DC, possibly indicative of their less pro-inflammatory phenotype. The more tolerogenic phenotype of the RA-DC was further supported by a reduced expression of TNF and NFκB1 mRNA in RA-DC. In addition, the RA-DC had upregulated CCR7 surface expression, in line with previous findings (22). CCR7 is important for CD103^+^ DC migration to the MLN (4).

In relation to chemokine gene expression, majority of chemokines were downregulated in RA-DC. CCL2 and CCL7, which are CCR2 ligands, are consistently higher both at protein and gene expression level in RA-DC than in Mo-DC. In the gut, CCL2 is important to attract CCR2 expressing mononuclear phagocytes (23). We hypothesize that the RA-DC-secreted CCL2 might contribute in the replenishment of phagocytic cells in the gut. Since CCR2, at least at the gene expression level, is downregulated in RA-DC, we do not consider an autocrine effect of CCL2 or CCL7 on the RA-DC very likely. Still, these chemokines might be of importance in this environment with a high microbial load. CCL2 and CCL7 have been shown to regulate cytokine production during infection in the lung and thereby contributing to infection control and host resistance (24).

To further explore the different characteristics of the RA-DC and Mo-DC, we investigated their response in an inflammatory setting. LPS induced expression of multiple genes, mainly in Mo-DC. Also, the fold upregulation of gene expression in the Mo-DC was much stronger than in RA-DC, giving additional support to a more tolerogenic profile of the RA-DC. In addition, we investigated the response of the DC types in an inflammatory setting. Although *L. reuteri*-CFS was just as potent as LPS in inducing IL10, IL6, and IL23 in both types of DC, the phylogenic tree of the clustogram revealed that there was a clear distinction between *L. reuteri*-CFS and LPS in regulating the expression of many DC genes.

IL10 is believed to be a key mediator of a regulatory environment. In IL10-deficient (25) or IL10 receptor-deficient mice (26), the animals develop severe colitis pathology following microbial colonization of the gut (27), strongly arguing for a regulatory role of IL10 in the gut. Also, *in vivo* administration of the same *L. reuteri* strain that we used in our study – *L. reuteri* DSM 17938 – increased IL10 mRNA expression in mice (18). Our study support this finding and further indicates that it might have been a secreted (postbiotic) factor from *L. reuteri* 17938 that played a role in inducing IL10 production *in vivo*. Indeed, studies on the beneficial effect of secreted factors from different lactobacilli on the host immune response are now being reported (20). Further support for a beneficial postbiotic effect is a very recent report that clearly demonstrates that a heat-killed probiotic *Lactobacillus* strain induced IL10 in peripheral immune cells by specifically stabilizing IL-10 mRNA levels, while this was not seen with live bacteria of the same strain (28). It has previously been shown that IL10 dampens IL23 production (29). Here however, IL23 is also upregulated in both RA-DC and Mo-DC after *L. reuteri* stimulation. This might appear contradictory to lactobacilli-mediated immune regulation as IL23 is mainly linked to the development of colitis (30). The downstream signal of IL23 contributes to the production of IL6 and IL17 (31), but it can also contribute to the production of IL22, another cytokine of the IL10 family (32). IL22 in the gut contributes to the maintenance of epithelial barrier and the induction of antimicrobial peptides, such as defensins and lectins (33, 34). In addition, IL23 can mitigate deleterious IL12 production by DC and thereby indirectly control excessive IFN-γ production by Th1 cells (32), suggesting that this IL23 can exert anti-inflammatory effects as well under certain circumstances. We also noted a prominent IL6 production after *L. reuteri* stimulation. Given its pleiotropic activity, IL6 can act pro-inflammatory but also contributes to intestinal homeostasis. For instance, it plays a major role in microbe-induced IgA production and maintenance of Th17 cell population in the gut (35, 36). Therefore, *L. reuteri*-CFS induced production of these cytokines could influence the micro-milieu of the gut, supporting the balance between tolerogenic responses to innocuous antigens and effective responses to infection.

*Lactobacillus reuteri*-CFS induced the production of several chemokines that are involved in gut homeostasis – CXCL1, CXCL5, CCL3, CCL15, and CCL20 in both RA-DC and Mo-DC. Local CXCL5 production regulates steady-state migration of neutrophils to the gut. CXCL5^−/−^ mice are devoid of neutrophils in the gut and as a consequence produce IL17 in an uncontrolled manner (37). CCL15 has been shown to function as an antimicrobial peptide in steady state conditions in the gut (38). CXCL1, apart from being important component of the intestinal immune response, is vital in restoring mucosal barrier integrity in murine models (39). In the gut, epithelial cells are the main source of CCL3 and CCL20 to recruit mononuclear phagocytes (40). DC secreting CCL3 and CCL20 might serve as an additional source to keep the gut in homeostasis.

Two other genes were markedly affected by *L. reuteri* in both DC types, namely THBS1 and CSF2, which encode for thrombospondin1 and colony stimulating factor, respectively. Both factors are associated with anti-inflammatory activity. Thrombospondin 1 is an autocrine negative regulator of DC activation, by arresting cytokine production that can be induced by microbial stimulation (41). CSF2 is associated with triggering the production of regulatory molecules in DC (42, 43).

There were also notable differences in surface marker expression between the two DC types in relation to *L. reuteri*-CFS exposure. *L. reuteri* strongly increased the percentage of CD14^+^ cells in the RA-DC, although these cells already had a higher CD14^+^ percentage in the unstimulated condition. CD14 expression in DC is believed to associate with a tolerogenic phenotype (44), in agreement with the more regulatory role of RA-DC but also suggesting that *L. reuteri* is able to support this phenotype. How the CD14 expression is induced and how this contributes to a more regulatory function is not clear although histamine has been shown to induce CD14 expression in DC (45). Indeed, we have previously shown a low but detectable histamine production by *L. reuteri* DSM 17938 (21). We have also noticed that *L. reuteri*-CFS dampens DC-SIGN mainly on RA-DC. It has been shown that lactobacilli can act on DC through DC-SIGN to modulate IL10 production by T cells (46).

In order to reach the T cells in the MLN, DC upregulate CCR7, which enables them to migrate and encounter T cells at the appropriate site. Here, we observed that *L. reuteri*-CFS upregulates CCR7 expression on the surface of RA-DC. Both at gene and protein levels, molecules associated with antigen presentation and T-cell costimulation were down regulated in the RA-DC upon stimulation with *L. reuteri*-CFS, and often more prominently than in Mo-DC. Indeed, tolerogenic-DC express low amount of costimulatory molecules and produce high level of IL10 (47, 48). The insufficient stimulatory signal for T cells from these DC in turn favors T cell-differentiation to Treg (49).

TLR2 expression was upregulated in RA-DC stimulated with *L. reuteri*-CFS, while downregulated in Mo-DC. It has previously been shown that TLR2 downstream signaling potently induce retinal dehydrogenases (RALDH) expression, an enzyme that is involved in metabolizing vitamin A to RA (50). Upregulation of TLR2 expression could be a positive feedback mechanism by *L. reuteri*-CFS in potentiating RA-DC, which resembles phenotypically GALT-DC, to produce more RA.

The RA-DC were better inducers of FOXP3- and IL-10 expression within the Treg cell compartment than Mo-DC, in line with previous finding, showing that that RA can enhance TGFβ1 induced IL10 production and FOXP3 expression in T cells (4). Compared to our results, the effect of RA is significantly more profound on the percentage of FOXP3 cells in this other publication (4). This might be attributed to experimental setup differences. We have tested both autologous and allogeneic T cells in our DC:T cell cocultures and also extended culture times with very similar results as displayed here (data not shown). However, we have used the whole T cell population, instead of naive T cells, where a regulatory induction may be masked or even inhibited by the activated and memory T cells present.

In an experimental animal model of NEC, *L. reuteri* DSM 17938 increased the frequency of FOXP3^+^ Treg and ameliorated the severity of the disease (51). Here, we saw surprisingly little effects on the Treg compartment after *L. reuteri*-CFS stimulation, both in RA-DC and Mo-DC. This suggests that the probiotic *L. reuteri*-CFS has relative limited effect on the T cell compartment that are mediated via the DC, regardless of the type of DC at least *in vitro*. Maybe additional factors present in the gut microenvironment are needed for inducing the Treg responses reported in *in vivo* models.

In conclusion, we show the combined effect of *L. reuteri*-CFS and RA in modulating the response of DC. Our results further indicate that the signaling pathways that are modulated by *L. reuteri*-CFS in RA-DC are different from that in the Mo-DC, which further strengthens the distinct phenotype of these two DC types and the ability of *L. reuteri-*CFS to modulate the DC population in the gut to support a mucosal-like phenotype and function. The importance of identifying the biological active molecules in the supernatant could also contribute in the future to use it as postbiotic treatment. In addition, it will help to identify the underlying mechanism, which could be used to fine-tune our probiotics to fit to health benefits.

## Author Contributions

Conceived and designed the experiments: YH, KR, BR, and E-SE. Performed the experiments: YH, MN, and NV. Analyzed the data: YH and MN. Contributed reagents/materials/analysis tools: E-SE. Wrote the manuscript: YH and E-SE.

## Conflict of Interest Statement

The authors declare that the research was conducted in the absence of any commercial or financial relationships that could be construed as a potential conflict of interest.
