# Supplementary material for: Postbiotic Modulation of Retinoic Acid Imprinted Mucosal-like Dendritic Cells by Probiotic Lactobacillus reuteri 17938 In Vitro
Source: Front Immunol. 2016 Mar 17;7:96. doi: 10.3389/fimmu.2016.00096 (PMC4794487; doi:10.3389/fimmu.2016.00096)
Supplement: Supplementary file 1 [file DataSheet_1.docx]

**Supplementary Figure 1.**

**
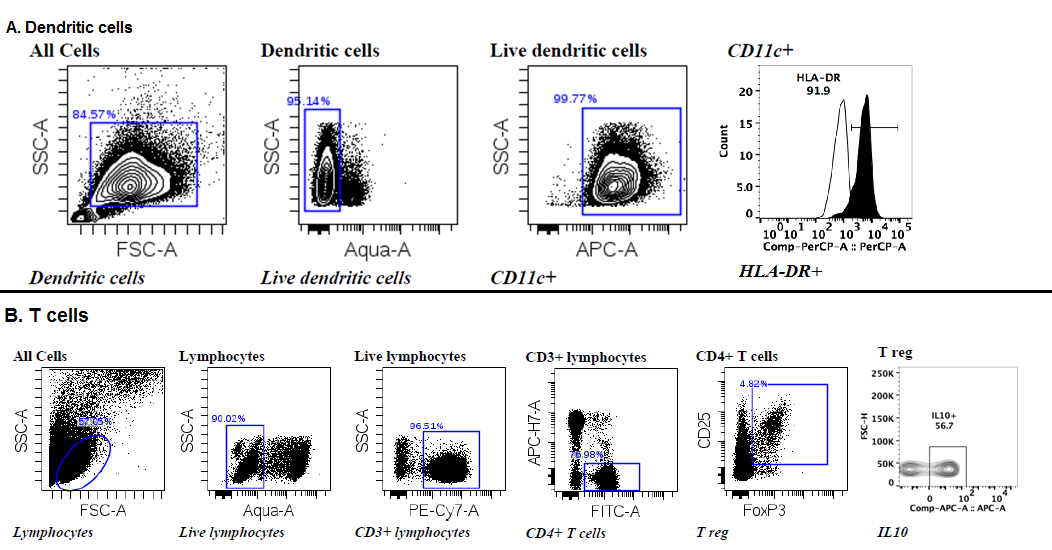
**

**Supplementary Figure 1. The gating strategy for DC (A) and T cells (B).**

**Supplementary Figure 2**


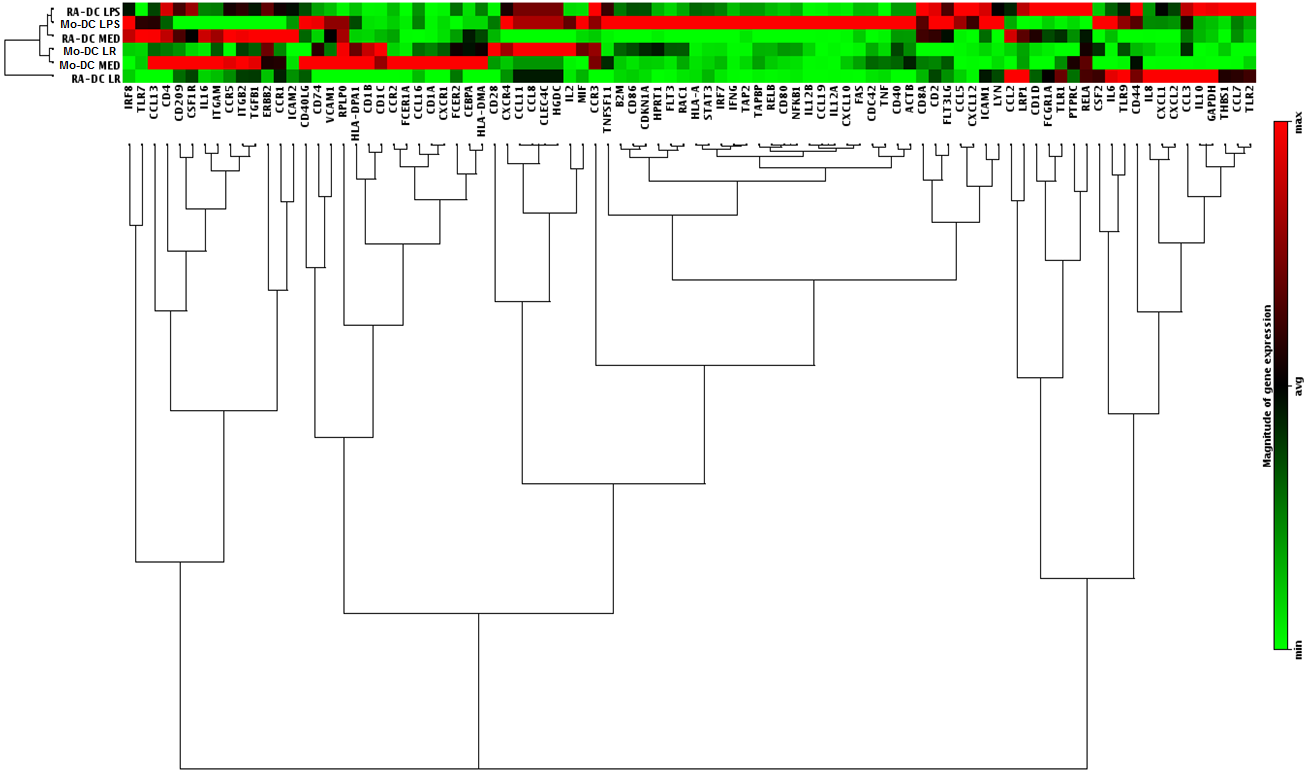


**Supplementary Figure 2. Cluster gram change in gene expression of RA-DC and Mo-DC, post-treatment**. RT^2^ PCR arrays were performed on the RNA extracted from the RA-DC and Mo-DC treated with *L. reuteri*-CFS or LPS or kept in culture medium for 24hrs. The clustergram was created based on non-supervised hierarchical clustering of the entire 84 genes dataset to show a heat map with dendrograms indicating co-regulated genes across dendritic cells and their treatment or individual dendritic cells treatments.

**Supplementary Figure 3**


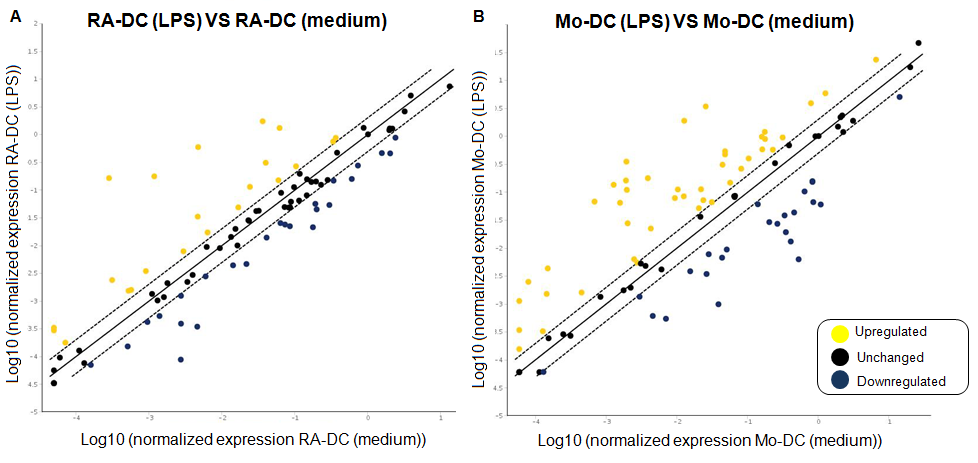


**Supplementary Figure 2. RA-DC and Mo-DC differentially regulate their genes in response to LPS**. RT^2^ PCR arrays were performed on the RNA extracted from the RA-DC and Mo-DC stimulated with LPS for 24hrs. The scatter plot illustrates the differences in gene expression of the LPS stimulated RA-DC and Mo-DC in comparison to culture medium. Up-regulated genes are shown in yellow, down-regulated genes are shown in blue and unchanged (<2 fold change) genes in black.

Supplementary Table I: Fold regulation of mRNA expression of RA-DC in medium compared to mRNA expression of Mo-DC in medium.

|  | *Name* | *Fold regulation* |  | *Name* | *Fold regulation* |  | *Name* | *Fold regulation* |
| --- | --- | --- | --- | --- | --- | --- | --- | --- |
| Cytokines | CCL11 | -1,06^B^ | **Cytokine receptors** | CCR1 | 1,61 | **Antigen presentation** | CD1A | **-103,87** |
|  | CCL13 | -1,01 |  | CCR2 | **-4,31^A^** |  | CD1B | **-8,98** |
|  | CCL16 | **-2,05** |  | CCR3 | -1,25 |  | CD1C | **-5,53** |
|  | CCL19 | **-3,01^A^** |  | CCR5 | 1,04 |  | CD1D | **42,30^A^** |
|  | CCL2 | **65,01** |  | CSF1R | -1,86 |  | CD209 | -1,46 |
|  | CCL3 | 1,91 |  | CXCR1 | **-12,36^A^** |  | CD28 | -1,14 |
|  | CCL5 | **5,40** |  | CXCR4 | **-3,60** |  | CD4 | -1,10 |
|  | CCL7 | **97,18^A^** |  | FLT3 | **-3,45^A^** |  | CD40 | -1,41 |
|  | CCL8 | -1,06^B^ |  | ERBB2 | 1,37 |  | CD40LG | **-2,17** |
|  | CSF2 | 1,00 | **Signal transduction** | CDKN1A | -1,96 |  | CD74 | **-5,65** |
|  | CXCL1 | **-4,05** |  | CEBPA | -1,94 |  | CD80 | **-9,30** |
|  | CXCL10 | **-7,66^A^** |  | CLEC4C | -1,06^B^ |  | CD86 | -1,52 |
|  | CXCL12 | -1,06^B^ |  | FAS | -1,31 |  | CD8A | 1,76 |
|  | CXCL2 | **-2,96^A^** |  | IRF7 | **-4,25^A^** |  | HLA-A | -1,30 |
|  | FLT3LG | 1,21 |  | IRF8 | **2,25** |  | HLA-DMA | **-2,20** |
|  | IFNG | **-2,69** |  | ITGAM | -1,20 |  | HLA-DPA1 | **-6,40** |
|  | IL10 | **2,85** |  | ITGB2 | -1,02 |  | TAPBP | **-2,47** |
|  | IL12A | -1,06^B^ |  | LYN | -1,25 |  | THBS1 | **3,35^A^** |
|  | IL12B | **-5,57^A^** |  | NFKB1 | **-2,58** | **Other cell surface receptors** | CD2 | 1,61 |
|  | IL16 | -1,09 |  | PTPRC | -1,49 |  | FCER1A | **-134,12** |
|  | IL2 | -1,06^B^ |  | RELA | -1,48 |  | FCER2 | **-3,40** |
|  | IL6 | **8,71** |  | RELB | **-3,80** |  | FCGR1A | **47,92^A^** |
|  | CXCL8 | 1,57 |  | STAT3 | -1,44 |  | LRP1 | **2,85** |
|  | MIF | 1,04 | **Antigen uptake** | CD44 | **-2,46** |  | TLR1 | 1,66 |
|  | TGFB1 | 1,04 |  | CDC42 | -1,79 |  | TLR2 | 1,25 |
|  | TNF | **-3,09** |  | ICAM1 | **2,38** |  | TLR7 | **6,79^A^** |
|  | TNFSF11 | **-2,29** |  | ICAM2 | **4,39** |  | TLR9 | -1,03 |
|  |  |  |  | RAC1 | -1,93 |  | VCAM1 | -1,98 |
|  |  |  |  | TAP2 | -1,25 |  |  |  |

^A^ This gene’s average threshold cycle is relatively high (> 30) in either the control or the test sample, and is reasonably low in the other sample (< 30). These data mean that the gene’s expression is relatively low in one sample and reasonably detected in the other sample suggesting that the actual fold-change value is at least as large as the calculated and reported fold-change result.

^B^ This gene’s average threshold cycle is either not determined or greater than the defined cut-off (default 35), in both samples meaning that its expression was undetected, making this fold-change result erroneous and un-interpretable. Values in bold are considered biologically relevant difference (≥ 2 fold change).

Supplementary table II: the fold change in the MFI of the surface markers of RA-DC compared to that of the Mo-DC

|  | HLA-DR | CD83 | CD86 | DC-SIGN |
| --- | --- | --- | --- | --- |
| Mo-DC | 1,00 | 1,00 | 1,00 | 1,00 |
| RA-DC | 2,20 (0,64-4,29) | 1,84 (0,78-3,48) | 2,18 (1,14-4,92) | 0,97 (0,61-1,71) |

Average fold increase (min-max) in MFI of 8 experiments

Supplementary Table III: Fold regulation of mRNA expression DC stimulated by *L. reuteri* compared to mRNA expression of DC in medium

|  | *Name* | *Fold regulation RA-DC* | *Fold regulation Mo-DC* |  | *Name* | *Fold regulation RA-DC* | *Fold regulation Mo-DC* |  | *Name* | *Fold regulation*  *RA-DC* | *Fold regulation Mo-DC* |
| --- | --- | --- | --- | --- | --- | --- | --- | --- | --- | --- | --- |
| Cytokines | CCL11 | -1,40^B^ | 1,23^B^ | **Cytokine receptors** | CCR1 | **-3,12** | -1,87 | **Antigen presentation** | CD1A | **-28,05^A^** | **-4,10** |
|  | CCL13 | **-23,75** | **-2,88** |  | CCR2 | **-5,31** | **-6,80^A^** |  | CD1B | **-4,86** | -1,12 |
|  | CCL16 | -1,40^B^ | -1,57 |  | CCR3 | -1,66^A^ | 1,02^A^ |  | CD1C | **-23,10** | 1,25 |
|  | CCL19 | **-3,76** | **-16,85^A^** |  | CCR5 | **-4,50** | **-5,60** |  | CD1D | **-2,83** | **5,37** |
|  | CCL2 | -1,45 | **6,85** |  | CSF1R | **-2,10** | **-7,28** |  | CD209 | **-2,99** | **-3,35** |
|  | CCL3 | 1,47 | **2,44** |  | CXCR1 | **-4,92** | **-2,96** |  | CD28 | **-2,39** | **2,70^A^** |
|  | CCL5 | **2,25** | **3,04** |  | CXCR4 | **3,78** | **5,76** |  | CD4 | **-5,43** | **-2,18** |
|  | CCL7 | 1,68 | -1,26 |  | FLT3 | -1,18 | **5,33** |  | CD40 | **-6,32** | 1,16 |
|  | CCL8 | -1,40^B^ | 1,23^B^ |  | ERBB2 | **-3,43** | 1,13 |  | CD40LG | -1,55 | **-4,33** |
|  | CSF2 | **14,03** | **15,19** | **Signal transduction** | CDKN1A | 1,01 | **2,67** |  | CD74 | **-2,83** | -1,62 |
|  | CXCL1 | **1351,18^A^** | **18,32^A^** |  | CEBPA | **-6,82** | -1,87 |  | CD80 | 1,89 | **2,69** |
|  | CXCL10 | **-8,00** | **-51,79^A^** |  | CLEC4C | -1,40^B^ | 1,23^B^ |  | CD86 | 1,26 | **3,17** |
|  | CXCL12 | -1,40^B^ | 1,23^B^ |  | FAS | **-2,58** | -1,90 |  | CD8A | **-3,68** | **-2,09** |
|  | CXCL2 | **464,65^A^** | **8,73** |  | IRF7 | 1,23 | -1,71 |  | HLA-A | -1,18 | -1,16 |
|  | FLT3LG | **-2,30** | -1,86^A^ |  | IRF8 | **-3,18** | 1,13 |  | HLA-DMA | **-4,89** | -1,69 |
|  | IFNG | -1,09 | -1,99 |  | ITGAM | **-5,90** | **-2,45** |  | HLA-DPA1 | **-3,32** | -1,38 |
|  | IL10 | **3,14** | -1,08^A^ |  | ITGB2 | **-3,05** | -1,95 |  | TAPBP | -1,36 | -1,14 |
|  | IL12A | -1,40^B^ | 1,23^B^ |  | LYN | 1,55 | -1,12 |  | THBS1 | **81,57** | **75,34^A^** |
|  | IL12B | 1,09 | **17,09** |  | NFKB1 | 1,22 | **2,45** | **Other cell surface receptors** | CD2 | **-2,11** | -1,66^A^ |
|  | IL16 | **-6,59** | **-5,56** |  | PTPRC | -1,46 | **-2,21^A^** |  | FCER1A | -1,65 | **-28,34** |
|  | IL2 | -1,40^B^ | **3,99** |  | RELA | -1,14 | -1,25 |  | FCER2 | -1,30 | -1,70 |
|  | IL6 | **4,41** | 1,71 |  | RELB | -1,25 | 1,32 |  | FCGR1A | -1,12 | **5,08** |
|  | CXCL8 | **266,87** | **67,43** |  | STAT3 | 1,09 | -1,10 |  | LRP1 | -1,12 | -1,04 |
|  | MIF | -1,54 | 1,38 | **Antigen uptake** | CD44 | **2,04** | -1,73 |  | TLR1 | 1,02 | **-4,67** |
|  | TGFB1 | **-3,32** | **-2,27** |  | CDC42 | -1,41 | -1,26 |  | TLR2 | **3,39** | **-6,80** |
|  | TNF | -1,15 | **-2,21** |  | ICAM1 | -1,21 | 1,36 |  | TLR7 | **-27,86^A^** | -1,13 |
|  | TNFSF11 | -1,69 | **-2,12** |  | ICAM2 | **-4,47** | -1,26 |  | TLR9 | **2,27** | 1,83 |
|  |  |  |  |  | RAC1 | -1,27 | 1,47 |  | VCAM1 | **-77,71^A^** | **-3,67^A^** |
|  |  |  |  |  | TAP2 | **-2,75** | -1,86 |  |  |  |  |

^A^ This gene’s average threshold cycle is relatively high (> 30) in either the control or the test sample, and is reasonably low in the other sample (< 30). These data mean that the gene’s expression is relatively low in one sample and reasonably detected in the other sample suggesting that the actual fold-change value is at least as large as the calculated and reported fold-change result.

^B^ This gene’s average threshold cycle is either not determined or greater than the defined cut-off (default 35), in both samples meaning that its expression was undetected, making this fold-change result erroneous and un-interpretable. Values in bold are considered biologically relevant difference (≥ 2 fold change).

Supplementary Table IV: the fold change in the MFI of the surface markers of RA-DC and Mo-DC post-stimulation

|  |  | HLA-DR | CD83 | CD86 | DC-SIGN |
| --- | --- | --- | --- | --- | --- |
| RA-DC | medium | 1,00 | 1,00 | 1,00 | 1,00 |
|  | *L. reuteri* | 0,95 (0,68-1,33) | 0,89 (0,51-1,38) | 1,73 (1,16-2,79) | 0,34 (0,22-0,40) |
|  | LPS | 1,8 (0,95-3,21) | 2,19 (0,80-5,48) | 2,51 (1,37-3,89) | 0,53 (0,31-0,79) |
|  |  |  |  |  |  |
| Mo-DC | medium | 1,00 | 1,00 | 1,00 | 1,00 |
|  | *L. reuteri* | 2,2 (1,01-4,39) | 3,2 (0,44-12,92) | 3,35 (1,00-5,21) | 0,72 (0,31-1,02) |
|  | LPS | 3,21 (1,30-5,35) | 7,45 (1,14-23,58) | 4,05 (1,44-5,91) | 0,80 (0,58-1,07) |

Average fold increase (min-max) in MFI of 4 experiments for RA-DC and 6 experiments for Mo-DC
